# Annotation of the human cerebrospinal fluid lipidome using high resolution mass spectrometry and a dedicated data processing workflow

**DOI:** 10.1007/s11306-016-1023-8

**Published:** 2016-04-07

**Authors:** Alexandre Seyer, Samia Boudah, Simon Broudin, Christophe Junot, Benoit Colsch

**Affiliations:** Profilomic SA, Boulogne-Billancourt, France; CEA-Saclay, DSV/iBiTec-S/SPI, Laboratoire d’étude du Métabolisme des Médicaments, MetaboHUB-Paris, Gif-Sur-Yvette, France

**Keywords:** Lipidomics, High resolution mass spectrometry, Neurological disorders, Biomarker discovery, Cerebrospinal fluid, Bioinformatics

## Abstract

**Introduction:**

Due to its proximity with the brain, cerebrospinal fluid (CSF) could be a medium of choice for the discovery of biomarkers of neurological and psychiatric diseases using untargeted analytical approaches.

**Objectives:**

This study explored the CSF lipidome in order to generate a robust mass spectral database using an untargeted lipidomic approach.

**Methods:**

Cerebrospinal fluid samples from 45 individuals were analyzed by liquid chromatography coupled to high-resolution mass spectrometry method (LC-HRMS). A dedicated data processing workflow was implemented using XCMS software and adapted filters to select reliable features. In addition, an automatic annotation using an in silico lipid database and several MS/MS experiments were performed to identify CSF lipid species.

**Results:**

Using this complete workflow, 771 analytically relevant monoisotopic lipid species corresponding to 550 unique lipids which represent five major lipid families (i.e., free fatty acids, sphingolipids, glycerophospholipids, glycerolipids, and sterol lipids) were detected and annotated. In addition, MS/MS experiments enabled to improve the annotation of 304 lipid species. Thanks to LC-HRMS, it was possible to discriminate between isobaric and also isomeric lipid species; and interestingly, our study showed that isobaric ions represent about 50 % of the total annotated lipid species in the human CSF.

**Conclusion:**

This work provides an extensive LC/HRMS database of the human CSF lipidome which constitutes a relevant foundation for future studies aimed at finding biomarkers of neurological disorders.

**Electronic supplementary material:**

The online version of this article (doi:10.1007/s11306-016-1023-8) contains supplementary material, which is available to authorized users.

## Introduction

Lipids are essential cellular constituents that have many critical roles in cellular functions. They are involved in energy storage, cell signaling as second messengers, and are major constituents of cell plasma membranes including lipid rafts (Simons and Toomre 2000). Their crucial role is highlighted by their involvement in a large number of heterogeneous diseases such as cancer, diabetes, neurological disorders and inherited metabolic diseases (Wenk 2005; Lamari et al. 2013). Abnormal concentrations of lipids are especially observed in various neurological disorders, including neurodegenerative conditions such as Alzheimer’s or Parkinson’s diseases and neurometabolic disorders (Han et al. 2002; Adibhatla et al. 2006; Colsch et al. 2008; Ariga et al. 2008; Haughey et al. 2010).

Due to the high structural diversity of lipid species arising from various combinations of fatty acyls and functional headgroups, the presence of isomeric and isobaric (i.e., species having the same nominal mass but distinct exact masses) lipid species, and their occurrence at a large concentration scale, a complete lipidomic profiling of biological matrices remains a challenge. The first analysis of complex lipid mixtures by mass spectrometry (MS) was introduced in 1990s by Han and Gross (1994). Since that time, improvements in MS instrumentation in terms of mass resolution, mass accuracy and duty cycles have expanded research in the field of lipidomics. In targeted approaches, the overall platform, i.e., sample preparation and MS detection is optimized for a predetermined number of lipid classes or sub-classes. These methods are mainly based on low resolution triple quadrupole (TQ) operated in precursor–ion scanning, neutral loss scanning or product ion modes. They offer high sensitivity and have successfully been applied to the lipid profiling of various biomaterials (Han and Cheng 2005; Han and Gross 2005; Quehenberger et al. 2010). A remarkable contribution in this field was the work of Quehenberger et al. (2010), who have quantified over 500 distinct molecular species distributed among the main lipid categories in plasma samples (Quehenberger et al. 2010). However, numerous targeted methods were necessary to achieve this broad lipidome coverage, thus limiting throughput capabilities of such approaches.

By enabling accurate mass measurements with sup-ppm errors, high resolution mass spectrometers (HRMS), which include Fourier transform ion cyclotron resonance (FTICR), Orbitrap, and Time-of-flight (TOF) instruments, have prompted the use of untargeted lipidomic approaches (Ejsing et al. 2009; Schwudke et al. 2011; Guo et al. 2012; Li et al. 2013;) through affording the possibility to separate isobaric lipid species. Using HRMS instruments, “shotgun approaches”, based on direct introduction of a total lipid extract into the mass spectrometer, have been developed for global lipidomic analysis and enable the measurement of several hundred of lipid species covering the 21 major lipid classes from yeast extracts (Ejsing et al. 2009). However, although these methods without prior chromatographic separation are fast and simple, their sensitivity is limited by major ion suppression effects and the lack of discrimination between isomeric lipid species (Han and Gross 2005).

As an alternative approach, liquid chromatography coupled to high-resolution mass spectrometry (LC-HRMS) can be used to improve the sensitivity (Bird et al. 2011; Junot et al. 2014) and facilitate the detection of minor lipid species. Furthermore, LC also enhances the separation of isomeric lipid species (Lee et al. 2011; Bird et al. 2012). These LC-HRMS-based global methods enable to achieve a broad lipidome coverage with detection and relative quantification of several hundred unique lipid species in a single experiment (Gregory et al. 2013).

However, dedicated informatic tools are required in order to handle of the huge quantity of data generated by LC/HRMS systems. Before automatic detection and annotation, raw data must be converted into data formats compatible with peak detection and alignment software tools such as MZmine (Katajamaa et al. 2006) or XCMS (Smith et al. 2012). Thanks to accurate mass measurements provided by HRMS, lipidomic features can be annotated by using lipid databases such as that of the LIPID MAPS consortium (Fahy et al. 2005), which introduced the “Comprehensive Classification System for Lipids”. This classification system aims to catalog lipid species and makes available online tools to support lipid identifications. More recently, the LipidBlast in silico tandem mass spectral database has been implemented and covers compounds of 26 lipid classes (Kind et al. 2013). In parallel, manufacturers have also developed commercial software tools, such as Lipid Search from ThermoFisher Scientific (Taguchi and Ishikawa 2010), Lipid View from AB Sciex (Ejsing et al. 2006) or SimLipid from Premier Biosoft, for direct annotation from raw data. However, reliable lipidomic data treatment workflows able to handle the detection and alignment of features, together with selection and annotation of analytically reliable ones, are still emerging.

Lipidomic profiles of human biological materials for biomarkers discovery are mostly performed in plasma (Quehenberger et al. 2010; Pizarro et al. 2013), cell or tissue extracts (Han and Cheng 2005; Ejsing et al. 2009), and to a lesser extent in urine (Touboul et al. 2005; Cui et al. 2008). Regarding neurological and psychiatric disorders, cerebrospinal fluid (CSF) could be a very informative fluid due to its constant physical contact with the brain. However, the total lipid content of human CSF appears to be low, with concentrations around 10–13 µg mL^−1^, according to previous studies performed on healthy individuals. This represents about 0.2 % of serum total lipid content (Irani 2009) with a huge heterogeneity of lipid species. For this reason, very few lipidomic studies have been conducted on CSF and most of them have been achieved using targeted methods focused on selected lipid families (Colsch et al. 2015), such as free fatty acids and their derivatives (Farias et al. 2011; Fonteh et al. 2014), glycerophospholipids, sphingomyelins, sterols (Tourtellotte 1959; Illingworth and Glover 1971) and phospholipid families (Mudler et al. 2003; Kosicek et al. 2010; Kosicek et al. 2012; Fonteh et al. 2013).

In this context, the objective of this work was to develop and validate an untargeted lipidomic approach by using UHPLC/HRMS, to implement a dedicated data processing workflow and to perform complementary MS/MS experiments in order to provide an extensive annotation of human CSF lipidome.

## Materials and methods

### CSF samples

CSF samples from 43 subjects including 35 patients with unknown neurological disturbances and eight patients without observed neurological signs (control CSF samples) were collected by Drs. F. Sedel and F. Lamari of the Pitie-Salpetriere Hospital (Paris, France) in the frame of the SPECMET 2 study (PHRC Grant number AOM 10300), with the agreement of the local ethics committee for clinical research studies. This cohort was designed in order to obtain the most representative structural diversity of lipid species in CSF samples. A quality control (QC) sample consisting of a mixture of equal aliquots of all samples included in this study was injected every ten samples. These QC samples were extracted and then injected in triplicate after successive dilutions from 2 to 8 at the beginning of the running sequence after blank series with and without internal standards in order to check the performances of the analytical system and to validate the reliability of the detected features.

### Reagents

LC–MS grade water (H_2_O) and methanol (MeOH) were from VWR International (Plainview, NY). HPLC grade ammonium formate, chloroform, 2-propanol (IPA) and formic acid were from Sigma-Aldrich (Sigma Chemical Co., St Louis, MO, USA). All internal standards used in this study (see Table S1 of the supplemental data) were purchased from Avanti Polar Lipids, Inc (Alabaster, AL).

### Sample preparation

CSF lipids were extracted according to a modified Bligh and Dyer method (Bligh and Dyer 1959). Briefly, 100 μL of CSF was added to 490 μL of chloroform/methanol 50:50 (v/v) and 10 μL of internal standard mixture (IS; see Table S1 in supplementary material). Samples were vortexed for 60 s and then sonicated for 30 s using a sonication probe. Extraction was performed after 2 h at +4 °C with mixing. In addition, 75 μL of ultra-pure water was added and samples were vortexed for 60 s before centrifugation at 4000 rpm for 10 min at 4 °C. The upper phase (aqueous phase), containing ganglioside species and several lysophospholipids, was transferred into a glass tube and then dried under a stream of nitrogen. The interphase which consists on a protein disk was discarded and the lower rich-lipid phase (organic phase) was pooled with the dried upper phase. Samples were then reconstituted with 100 μL of MeOH/IPA/H_2_O 65:35:5 (v/v/v), vortexed for 30 s and sonicated for 60 s before injection.

### Liquid chromatography

The reversed phase chromatographic conditions were adapted from Hu et al. (2008). Methanol was preferred to acetonitrile since it has been observed that it decreased the carryover of most apolar lipid species (data not shown). CSF total lipid extracts were separated on an Dionex Ultimate 3000 UPLC system (Thermo Scientific, San Jose, CA) using a kinetex C_8_ 150 × 2.1 mm, 2.6 μm column (Phenomenex, Sydney, NSW, Australia). Mobile phase A consisted of H_2_O/MeOH 60/40 (v/v) and 0.1 % formic acid and mobile phase B of IPA/MeOH 90/10 (v/v) and 0.1 % formic acid. Ammonium formate (10 mM) was added to both mobile phases in the positive ion mode. The presence of ammonium salt in mobile phases decrease drastically the detection of sodium adducts which now constitute minor lipid species in the positive ion mode (data not shown). The gradient program was as follows: solvent B was maintained for 2.5 min at 32 %, from 2.5 to 3.5 min it was increased to 45 % B, from 3.5 to 5 min to 52 % B, from 5 to 7 min to 58 % B, from 7 to 10 min to 66 % B, from 10 to 12 min to 70 % B, from 12 to 15 min to 75 % B, from 15 to 19 min to 80 % B, from 19 to 22 min to 85 % B, and from 22 to 23 min to 95 % B; from 23 to 25 min, 95 % B was maintained; from 25 to 26 min solvent B was decreased to 32 % and then maintained for 4 min for column re-equilibration. The flow rate was 400 μL/min and the column temperature was set to 60 °C.

### Mass spectrometry

After injection of 10 μL of sample, the column effluent was directly introduced into the heated Electrospray (HESI) source of a Q-Exactive mass spectrometer (Thermo Scientific, San Jose, CA) and analysis was performed in both ionization modes in two separate runs. The HESI source parameters were as follows: the spray voltage was set to 3.7 kV and −3.1 kV in positive and negative ionization mode, respectively. The heated capillary was kept at 360 °C and the sheath and auxiliary gas flow were set to 15 and 10 (arbitrary units), respectively. Mass spectra were recorded in full-scan MS mode from *m/z* 50 to *m/z* 2000 at a mass resolution of 140 k, full width at half-maximum (FWHM) at *m/z* 200, and at a scan speed of 2 Hz. External mass calibration was performed before analysis.

After LC-HRMS analysis of samples and annotation of features, QC samples were re-injected for higher energy collisional dissociation (HCD) MS/MS experiments in positive and negative ion modes on the same instrument set in targeted mode using inclusion lists. The isolation width was set at *m/z* 1.5, the normalized collision energy was 25 % and the mass resolution was set at 17,500 FWHM at *m/z* 200. HCD mass spectra were inspected manually in order to confirm annotations.

### Data processing

Raw files were first of all converted to mzXML format with MSconvert (ProteoWizard). Peak detection, alignment and integration were performed with the XCMS software package version 1.30.3 (Smith et al. 2006) including the CentWave algorithm (Tautenhahn et al. 2008). The list of the parameters used is available as supplementary material (see Table S2 provided as supplementary material). A data matrix was generated by the software. It contained the list of all detected features, including information such as accurate measured masses, retention times and areas of chromatographic peaks.

## Results and discussion

We first of all report on the development and validation of a lipidomic method based on reversed phase liquid chromatography coupled to high-resolution mass spectrometry (RPLC-HRMS). A data processing workflow was also developed, including automatic peak detection and alignment using the XCMS package (Tautenhahn et al. 2008; Smith et al. 2012), several filtration steps in order to obtain a list of reliable features, and automatic feature annotation which was achieved thanks to an in silico lipid database developed in our laboratory (see Table S5 in supplementary material) and based on accurate measured masses, retention time windows and relative isotopic abundance (RIA) of lipid species. This lipidomic approach was then used to analyze CSF samples collected from 43 individuals. Finally, MS/MS experiments were performed in order to improve lipid annotations.

### Method development and validation

The eight lipid families established by the LIPID MAPS Consortium represent a broad diversity of structure (Fahy et al. 2009). As a consequence, the efficiency of any untargeted lipidomic study rely on (i) the completeness and reliability of lipid extraction, (ii) the versatility and the sensitivity of the LC/MS profiling method, and on (iii) associated data processing tools that are mandatory to handle a huge amount of data.

#### Extraction recovery and repeatability

Using a modified Bligh and Dyer extraction conventionally used to analyze major lipid families in biological matrices, recoveries were calculated from CSF QC samples (n = 5) spiked with 22 non endogenous internal standards (IS) representing the five major lipid families (fatty acyls, glycerophospholipids, glycerolipids, sphingolipids and sterol lipids, see Table S1 provided as supplementary material). Their corresponding chromatographic peak areas were compared to those obtained from CSF extracts spiked with the same internal standard mixture after extraction. Using the described method, mean recoveries for the five lipid families were all above 80 % (see Table S3 provided as supplementary material): i.e., 88.2 % for fatty acyls, 90.6 % for sphingolipids, 80.6 % for glycerophospholipids and 88.7 % for glycerolipids.

#### Assessment of the lipidome coverage provided by the RPLC-HRMS method

**Fig. 1:**
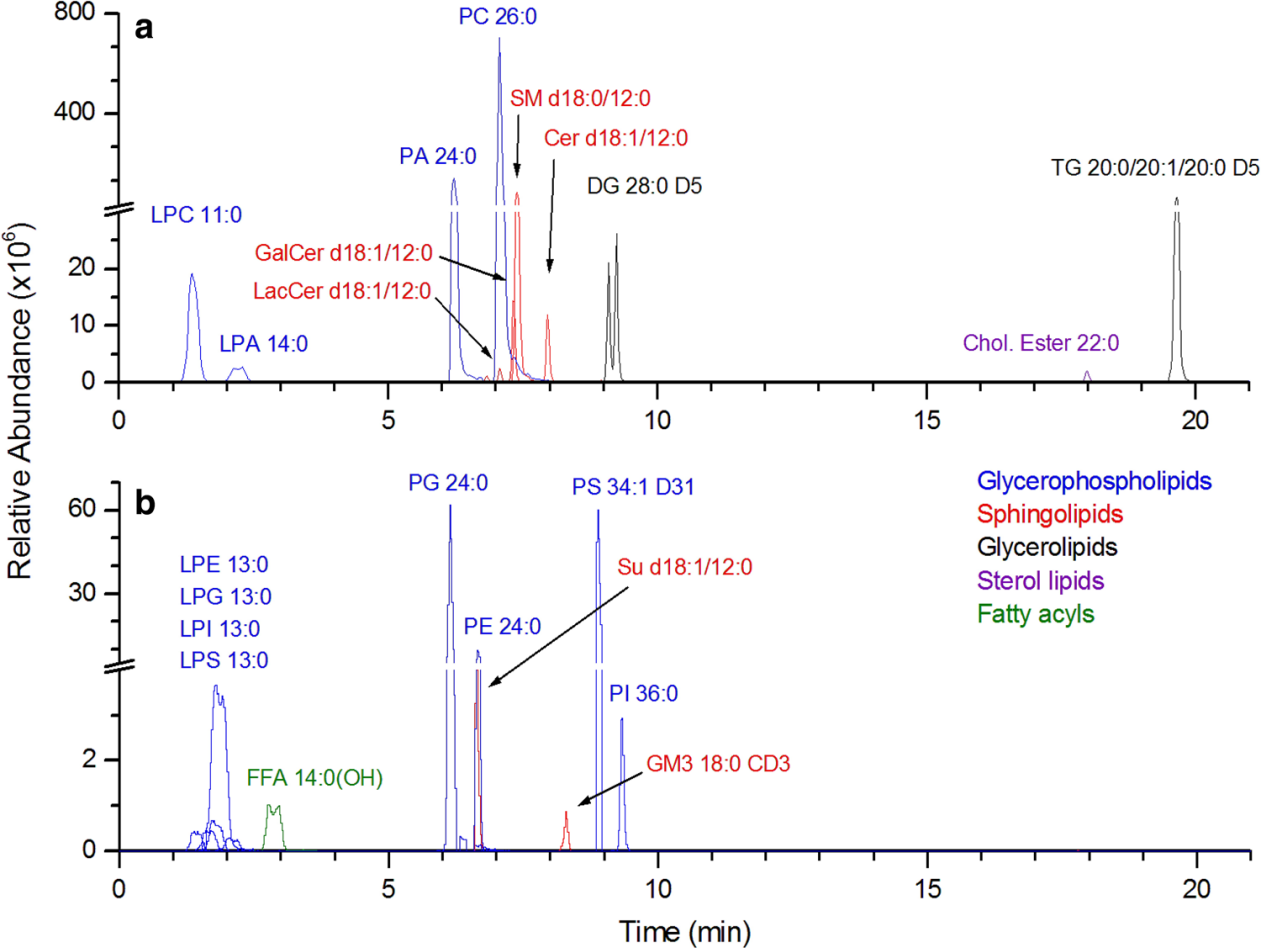
Extracted ion chromatograms of the 22 internal standards detected either in the positive (**a**) or in the negative (**b**) ion mode

Extracted ion chromatograms of IS spiked in CSF are displayed in Fig. 1. Both positive (Fig. 1a) and negative (Fig. 1b) electrospray ionization modes were used. As expected, the addition of ammonium formate in the positive ion mode mobile phases enhanced the signal of neutral lipid species (sterols, cholesteryl esters, MG, DG and TG internal standards) and phosphatidic acids (PA and LPA), which were detected as [M + NH_4_]^+^ adducts (Murphy et al. 2001). Thereby, the phosphatidylcholine (PC) internal standard including its associated lyso-forms (LPC), ceramides (Cer), galactosylceramides (GalCer), lactosylceramides (LacCer), and sphingomyelins (SM) were detected as [M + H]^+^ ions, whereas the free fatty acids, phosphatidylethanolamine (PE), phosphatidylinositol (PI), phosphatidylglycerol (PG), phosphatidylserine (PS) and their lyso forms (LPE, LPI, LPG and LPS, respectively), sulfatides (Su) and monosialo-gangliosides (SGL) were detected as [M − H]^−^ ions. Despite of the use of ammonium formate, sodium adducts ([M + Na]^+^) were detected for some lipid classes, i.e., Cer, HexCer, SM, LPC, PC, glycerolipids (DG and TG) and cholesteryl esters. Choline containing lipid classes (SM, LPC and PC) were also detected in the negative ion mode as formate adducts ([M − H + CHO_2_]^−^). Finally, water losses in the positive ion mode ([M + H − H_2_O]^+^) were observed for Cer, HexCer and DG lipid standards, as already described (Christie and Han, 2010). All these pseudo-molecular, adducts ions and in source fragments were added in the in silico database to improve annotation confidence (see Table S5 in supplementary material).

#### Validation of the RPLC-HRMS method

To assess its ability to detect changes in lipid profiles for clinical application and biomarker discovery, the developed method was validated by intra-assay precision and linearity studies performed on the internal standard mixture.

The intra-assay precision (regarding chromatographic peak areas and retention times) was evaluated by performing five independent extractions of the CSF pools spiked with the IS mixture (see Table S1, provided as supplementary material, for their concentrations). Relative standard deviations (RSDs) of chromatographic peak areas and retention times are shown in Table 1. Regarding peak areas, standard deviation ranged from 2.8 % to 20.1 % (average of 8.5 %). The lowest variations were obtained for the sphingolipid and fatty acyls families with an average RSD of 3.9 and 5.8 %, respectively, whereas the highest variations were found for neutral lipid families with 20.1 and 16.9 % for DG and TG species, respectively. In addition, retention time variations of all standards were also satisfactory with an average variation of 0.8 %.

**Table 1:** Evaluation of the repeatability, retention time variation and the linearity (from 10 to 1000 ng mL^−1^) of the 22 internal standards, representative of the five lipid categories

Lipid category	Repeatability (RSD, %)	Retention time variation (RSD, %)	Linearity *R*²
Free fatty acids			
C14:0(OH)	5.8	1.1	0.997
Sphingolipids			
d18:1/12:0 Cer	3.9	0.7	0.999
d18:1/12:0 GalCer	4.7	0.7	0.999
d18:1/12:0 lacCer	3.1	0.7	0.999
d18:1/12:0 Su	3.8	0.4	0.998
d18:0/12:0 SM	5.1	0.7	0.999
GM3 CD3	2.8	0.3	0.997
Glycerophospholipids			
11:0 LPC	10.6	1.7	0.992
14:0 LPA	18.5	1.9	0.979 (50–1000 ng mL^−1^)
13:0 LPE	6.5	0.8	0.995
13:0 LPS	5.8	2	0.997
13:0 LPI	10.3	1.7	0.979
13:0 LPG	7.6	1	0.989
26:0 PC	10.6	0.7	0.985
24:0 PA	8.9	0.7	0.998
24:0 PE	6.5	0.2	0.990
34:1 D31 PS	4.9	0.2	0.996
36:0 PI	7.8	0.4	0.986
24:0 PG	7.8	0.4	0.994
Glycerolipids			
1,3-28:0 D5 DG	20.1	0.7	0.974
60:1 D5 TG	16.9	0.5	0.935 (50–1000 ng mL^−1^)
Sterol lipids			
22:0 cholesteryl ester	14.8	0.5	0.981 (100–1000 ng mL^−1^)

CSF QC samples were also spiked with IS at five different concentrations ranging from 10 to 1000 ng.mL^−1^ (final concentrations, n = 3) in order to evaluate the linearity of the method (Table 1). Most of the standards were detected at the lowest concentration with regression coefficients (r^2^) ranging from 0.979 to 0.999. LPA and TG standards were detected from 50 ng.mL^−1^ with regression coefficients of 0.979 and 0.935, respectively. A particular behavior was observed for the cholesteryl ester internal standard, which was detected at 10 ng.mL^−1^, but whose linearity range started at 100 ng.mL^−1^.

#### Development of a data processing workflow

**Fig. 2:**
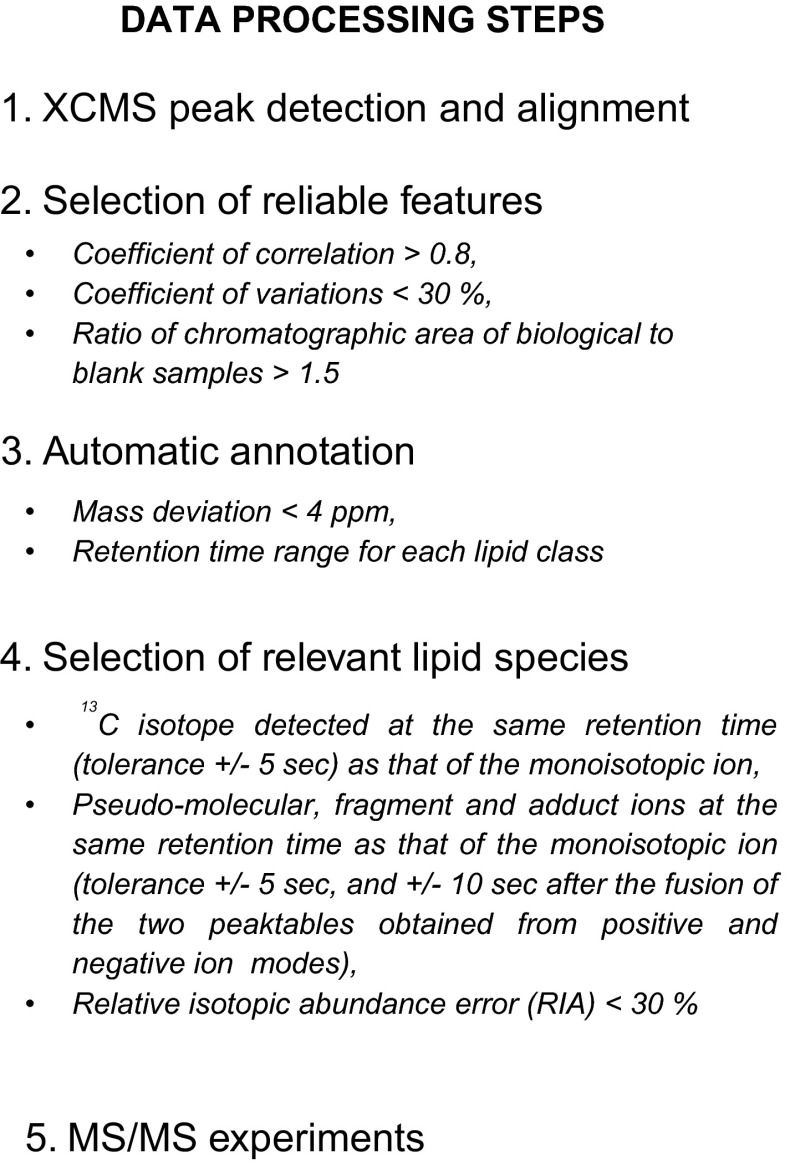
The data processing workflow. Features were filtered according to the following criteria: (i) the coefficients of correlation between dilution factors of QC samples (by factors of 1, 2, 4 and 8) and areas of chromatographic peaks (above 0.8), (ii) the coefficients of variation of areas of chromatographic peaks related to features detected in QC samples (less than 30 %) and (iii) the ratio of chromatographic areas of biological to blank samples (more than a factor of 1.5). Features were then annotated using a spectral database on the bases of exact masses and retention time windows specific to each lipid family. Each unique lipid species must be detected by XCMS/*CentWave* with at least its corresponding ^13^C isotope, and eventually fragment and adduct ions, which must be eluted at the same retention time as that of the pseudo molecular ion (with a tolerance of ±5 s, and ±10 s after the fusion of the two peaktables obtained in the positive and negative ion modes). Finally, each lipid species must have a RIA error below 30 %

From these complex high-resolution raw data, automatic detection of features (characterized by unique mass-to-charge ratio and retention time) is a crucial step and has to be reliable in order to detect as many ions as possible and minimize false positives. To this end, a data processing workflow including peak detection, selection of analytically relevant features and data set annotation was developed, as shown in Fig. 2.

Regarding peak detection, as preliminary works undertaken at our laboratory and using the *MatchedFilter* algorithm were not successful, we selected the already implemented *CentWave* algorithm, another peak detection algorithm (Tautenhahn et al. 2008). This algorithm is based on the concomitant detection of mass traces (named “regions of interest (ROI)”) and chromatographic peaks of different widths, which is relevant in the context of a non-targeted analysis of various lipid subclasses with different chromatographic peak shapes. The main *CentWave* parameters, i.e., the range of chromatographic peak width and the signal-to-noise threshold for validation of features, were carefully optimized by comparing false-negative and false-positive rates for manually annotated datasets.

The reliability of the XCMS software in term of peak integration was evaluated using chromatographic peaks related to internal standards and endogenous lipids from CSF samples spiked with the IS mixture. Areas of 164 chromatographic peaks (including 110 annotations corresponding to internal standard signals and the 54 endogenous lipids), ranging between four orders of magnitude, and automatically detected by XCMS were compared with those integrated by using Xcalibur (Thermo Fisher scientifics, Courtaboeuf, France) via the QuantBrowser module (ICIS algorithm). Results are shown in figure S1 in supplementary material. The linear regression analysis results were satisfactory with a coefficient of determination above 0.999 (r^2^ = 1.000 and r^2^ = 0.999 with only the 20 less intense ions) and a slope of 1.000, which indicates that automatic integration by XCMS performs well.

The XCMS output is a data matrix containing few thousands of features, either specific to the sample set, or corresponding to artifacts introduced by the LC–MS system or the sample preparation. In order to select analytically relevant features, a peak table filtration step was implemented according to the following criteria: (i) the coefficient of correlation between serial dilutions of QC samples (by factors of 1, 2, 4 and 8) and areas of the related chromatographic peaks should be above 0.8, (ii) the coefficients of variation of the areas of chromatographic peaks of features in QC samples should be less than 30 %, and (iii) the ratio of chromatographic area of biological to blank samples should be above a value of 1.5.

Feature annotation was then achieved through the implementation of an in silico database which contains 28,807 exact masses corresponding to pseudo-molecular ions ([M + H]^+^, [M − H]^−^ and [M − 2H]^2−^), adducts ([M + NH_4_]^+^, [M + Na]^+^, [M − H + CHO_2_]^−^), and in source fragments ([M + H − H_2_O]^+^) ions of the five major lipid families indexed in the LIPID MAPS library, along with their corresponding ^13^C and double ^13^C isotopes. Furthermore, specific retention time windows for each lipid class were also added by examining retention times of species containing the longest and the shortest fatty acyl chains (see Table S4 in supporting information). Thanks to scripts developed in R language, exact masses of XCMS detected features were matched to those of our in silico database (±4 ppm). Annotations were kept if the retention times of the corresponding features were within the retention time window specific of their putative lipid class.

However, accurate measured masses and retention time windows were not sufficient to assess the relevance of lipid species, and additional spectral information was required to confirm annotations (Kind and Fiehn 2006; Erve et al. 2009; Xu et al. 2010). Annotated lipid species were thus kept (i) if their ^13^C isotope was detected and aligned in time (±5 s) and (ii) if all annotated signals corresponding to the same lipid species (i.e., pseudo-molecular ions, adduct ions and/or in source fragments, either as monoisotopic or ^13^C and 2×^13^C isotopes) must have the same retention time as a reference ion (±5 s, and ±10 s between two ionization modes after merging corresponding peaktables).From the analysis of lipid standards spiked in CSF, we were able to determine these reference ions which correspond to the most intense ion of each lipid classes (i.e., pseudo-molecular ions or adduct ions, depending on the lipid class, see Table S5 in the supplementary material). In addition, the relative isotopic abundance (RIA) between the monoisotopic ion and its corresponding ^13^C isotope, were automatically calculated according to Eq. (1) and compared to theoretical ones. Annotated lipid species with a RIA error higher than 30 % were filtered out. This threshold of 30 % was selected since RIA errors of all internal standards were below this value (see figure S2 in supporting information).

1 $${\text{RIA error }}\left( {\% } \right) = 100 \times \frac{{{\text{RIAexp}} - {\text{RIAtheo}}}}{\text{RIAtheo}}$$

### Annotation of the human CSF lipidome

The RPLC-HRMS based lipidomic method and its associated data processing workflow were applied to the analysis of 43 human CSF samples. From complex LC/HRMS raw data, 51,449 features were generated in combined positive and negative ion modes by the XCMS/*Centwave* process, among which 32,478 were found to be analytically relevant (i.e., satisfaction values including dilution factors of coefficient variation between QC samples and ratio of chromatographic area of biological to blank samples). Then, 771 features were annotated, corresponding to pseudo-molecular, in-source fragment and adducts ions, as shown in Table 2. Of these annotations, 550 features were attributed to unique lipid species. This result emphasizes that many reliable features are not annotated by our in silico database and remain to be identified.

**Table 2:** Endogenous lipid species annotated in human CSF samples

Lipid category	Positive ion mode				Negative ion mode			Mean RSD (%; *n* = 8 QC samples)	Mean correlation coefficient	Mean absolute mass deviation (ppm)	Mean RIA absolute error (%)
	[M + H]^+^	[M + NH_4_]^+^	[M + Na]^+^	[M + H − H_2_O]^+^	[M − H]^−^	[M − 2H]^2−^	[M – H + CHO_2_]^−^				
Fatty acyls											
FA					9			6.31	0.975	2.615	3.55
PFAA	4		3					11.47	0.998	1.210	2.34
Sphingolipids											
Sphingoid bases	2							9.84	0.990	1.258	7.38
Cer	27		7	5	5			9.32	0.987	1.759	7.30
HexCer	14		4	6	2			13.34	0.993	0.745	4.13
Su					20			12.62	0.990	0.968	3.50
SM	28		12				24	12.99	0.991	1.196	6.53
Gangliosides					9	8		6.55	0.983	2.432	7.13
Glycerophospholipids											
LPC	16		3				10	11.92	0.995	1.184	5.88
LPE					4			6.44	0.978	2.214	6.54
LPS					2			13.88	1.000	0.438	1.91
LPI					5			12.39	0.996	2.231	3.84
LPG					3			13.30	0.999	1.527	2.27
PC	117		24				52	8.67	0.984	1.833	7.22
PA		7						5.99	0.976	2.236	8.00
PE					39			4.85	0.981	2.432	9.37
PS					46			12.75	0.990	0.981	5.74
PI					12			12.63	0.997	0.587	3.65
PG					5			11.10	0.990	0.890	10.12
Glycerolipids											
DG		35	14	9				6.83	0.977	2.334	9.37
TG	1	121	70					11.66	0.989	0.941	7.48
Sterolipids and steryl-esters	2	16	13	1				13.18	0.992	1.176	3.97

Endogenous lipid species were detected by ESI–MS under different ionic forms such as [M + H]^+^ and [M − H]^−^ ions, but also adduct ions such as [M + NH_4_] ^+^, [M + Na] ^+^, [M − H + CHO_2_]^−^ and in source-fragment ions [M + H − H_2_O]^+^, as observed for lipid standards. Interestingly, gangliosides were detected as singly charged [M − H]^–^ ions for monosialoganglioside species, and also as doubly charged [M − 2H]^2−^ ions with two to four attached sialic acid molecules. The five major lipid families were represented and annotated in both positive and negative ionization modes, including 13 fatty acyls (9 fatty acids and 4 fatty amides), 108 sphingolipids (2 sphingoïd bases, 27 ceramides, 14 hexosylceramides, 20 sulfatides, 28 sphingomyelins and 17 gangliosides), 256 glycerophospholipids (117 PC, 7 PA, 39 PE, 46 PS, 12 PI, 5 PG and their corresponding lyso-forms: 16 LPC, 4 LPE, 2 LPS, 5 LPI, 3 LPG), 156 glycerolipids (35 diacylglycerols and 121 triacylglycerols) and 17 sterols and steryl-esters (cholesterol and cholesteryl-esters).

Liquid chromatography coupled to ultra-high resolution mass spectrometry (*i.e.*, mass resolution power higher than 100 k, FWHM at *m/z* 200) was of special interest to distinguish isobaric and isomeric lipid species. Furthermore, MS/MS experiments were used to improve the annotation of lipid species.

#### Discrimination of isobaric lipid species

The presence of numerous isobaric lipid species is one the most challenging issue for a reliable lipidome annotation (Schwudke et al. 2011). Indeed, lipid species can be detected under different ionization forms and it is common to detect different lipid species belonging to different classes with the same nominal mass, but with distinct exact masses.

**Fig. 3:**
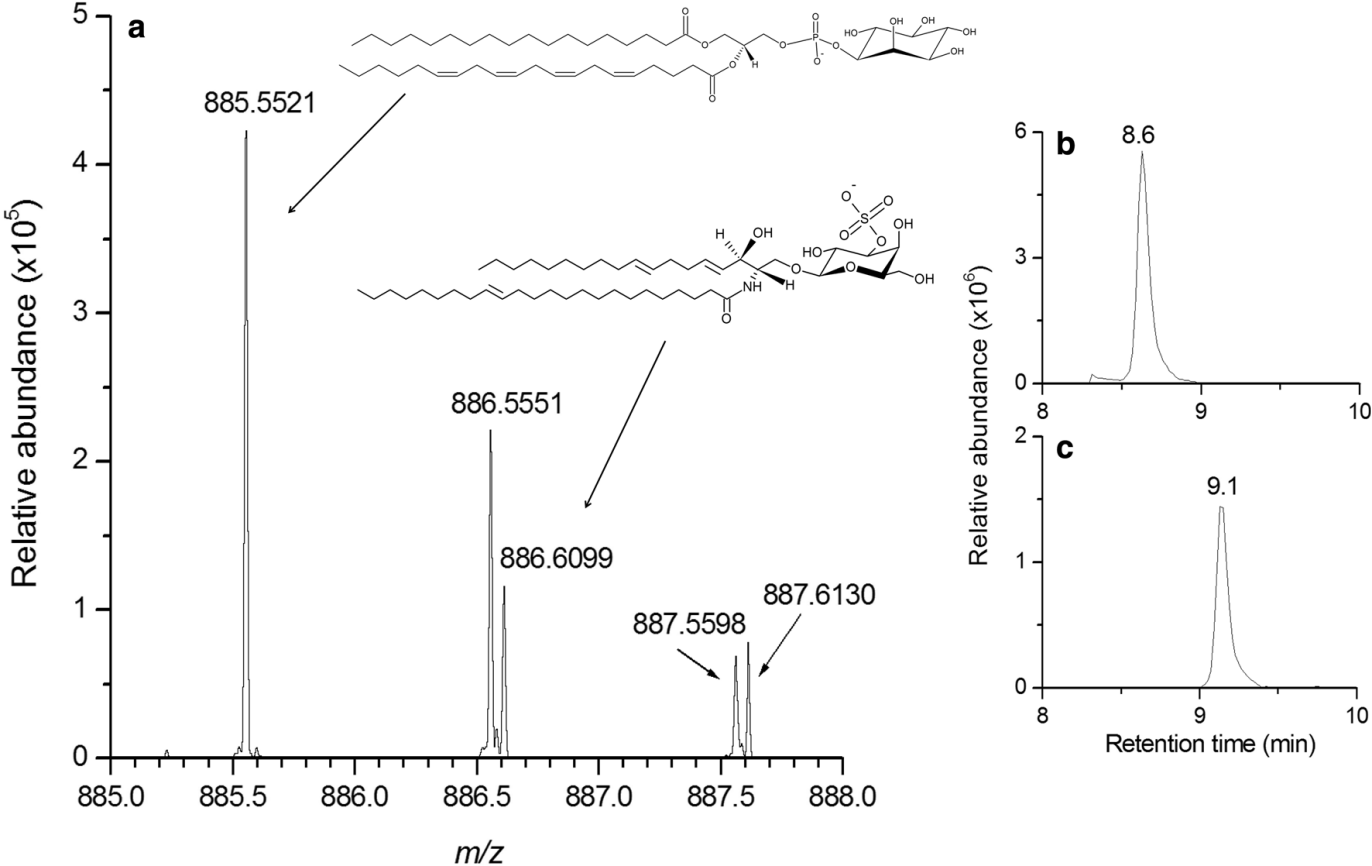
Extracted ion chromatogram between 8 and 10 min, obtained from detection in the negative ionization mode (**a**). Thanks to the implementation of an UHPLC method, two isobaric ions can be separated. The first one at 8.6 min (**b**) corresponds to the ^13^C isotope of PI38a:4, at *m/z* 886.5551, and the second one at 9.1 min (**c**) to the deprotonated ion of sulfatide d18:2/C24:1, at m/z 886.6099

As an example, an extracted ion chromatogram between 8 and 10 min is shown in Fig. 3a, with the simultaneous detection of the deprotonated ion [M − H]^−^ corresponding to the PI38a:4 at *m/z* 885.5521 and its ^13^C isotope at *m/z* 886.5551. Furthermore, another isobaric deprotonated [M-H]^−^ ion corresponding to the sulfatide lipid species (d18:2/C24:1) was also detected at *m/z* 886.6099. The use of UHPLC enabled to separate these two isobaric species, with retention times at 8.6 min for the PI (Fig. 3b) and 9.1 min for the sulfatide (Fig. 3c), respectively. The identity of the sulfatide lipid species was then confirmed by MS/MS experiments by the presence of the characteristic *m/z* 97.1044 ion corresponding to the sulfate group (see Table S4 provided as supplementary material) already found in mammalian brain (Colsch et al. 2004).

Our data showed that isobaric ions represent about 50 % of the 771 annotated lipid species in human CSF. This emphasizes the limit of using shotgun approaches and reinforces the relevance of using liquid chromatographic methods in complement to high resolution mass spectrometry.

#### MS/MS experiments improved the annotation of lipid species

The reliable characterization of the detected lipid species represents a critical issue for a global lipidomic approach. The implementation of our LC/HRMS method coupled to a data processing workflow enabled the annotation of 771 features corresponding to 550 unique lipid species in human CSF. MS/MS experiments were conducted on a QC sample using inclusion lists in both positive and negative ion modes in order to improve these annotations.

**Fig. 4:**
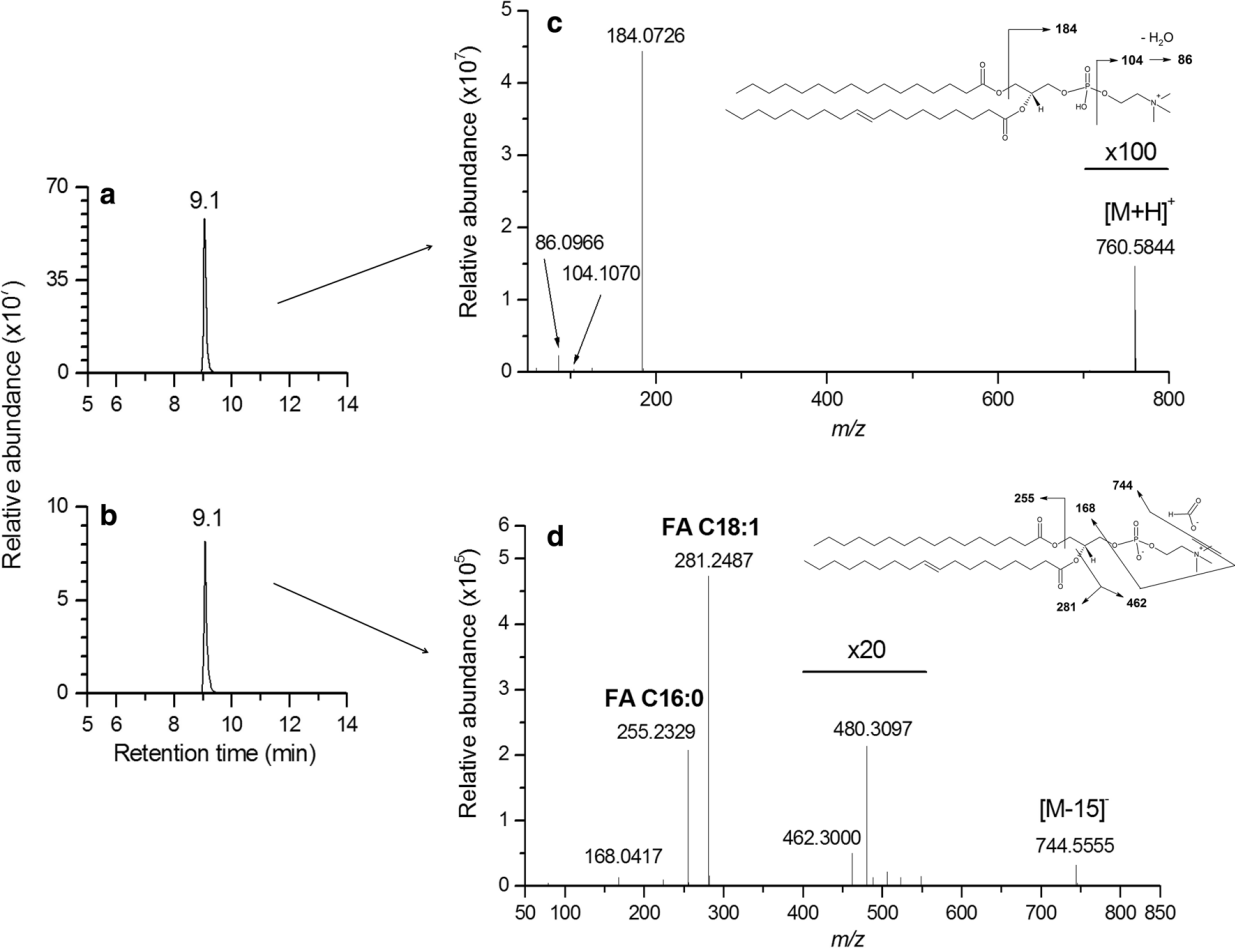
Extracted ion chromatograms of the PC34a:1 lipid species. The [M + H]^+^ ion at *m/z* 760.5844 (**a**), and the [M − H + CHO_2_]^−^ ion at *m/z* 804.5778 (**b**) were detected in the same retention time range (9.1 min ± 10 s), in the positive and negative ion mode, respectively. The CID mass spectrum of the protonated [M + H]^+^ ion confirmed the presence of the phosphocholine polar head (**c**), whereas the CID mass spectrum of the formate adduct [M − H + CHO_2_]^−^ ion enabled the detection of the two FA C18:1 (*m/z* 281.2487) and FA C16:0 (*m/z* 255.2329) associated fatty acids (**d**), leading to an improved structural characterization of this lipid species

The detection of some particular lipid species such as LPC/PC and SM in both positive and negative ionization modes, as protonated ion ([M + H]^+^) and formate adduct ([M − H + CHO_2_]^−^), enabled to confirm their annotation and also improved their structural characterization. This is for example displayed in Fig. 4 for PC34a:1. After having checked that all the ions related to this lipid species were eluted at the same retention time (at 9.1 min) in both positive and negative ion modes (Fig. 4a, b), MS/MS fragmentation of the [M + H]^+^ ion at *m/z* 760.5844 enabled to confirm the presence of the phosphocholine polar head thanks to the characteristic fragment ions at *m/z* 184.0726, *m/z* 104.1070 and *m/z* 86.0966 (Fig. 4c) (Murphy and Axelsen 2011). Furthermore, in negative ion mode, the fragmentation of the formate adduct ([M − H + CHO_2_]^−^) ion related to this PC34a:1 species led to an [M − 15]^−^ ion corresponding to a neutral loss of a methyl group (Harrison and Murphy 1995) detected at *m/z* 744.5555 and enable the detection of the two fatty acids FA C18:1 (*m/z* 281.2487) and FA C16:0 (*m/z* 255.2329) of this lipid species (Fig. 3d), thus confirming the annotation proposal.

#### MS/MS experiments enabled to distinguish isomeric lipid species

**Fig. 5:**
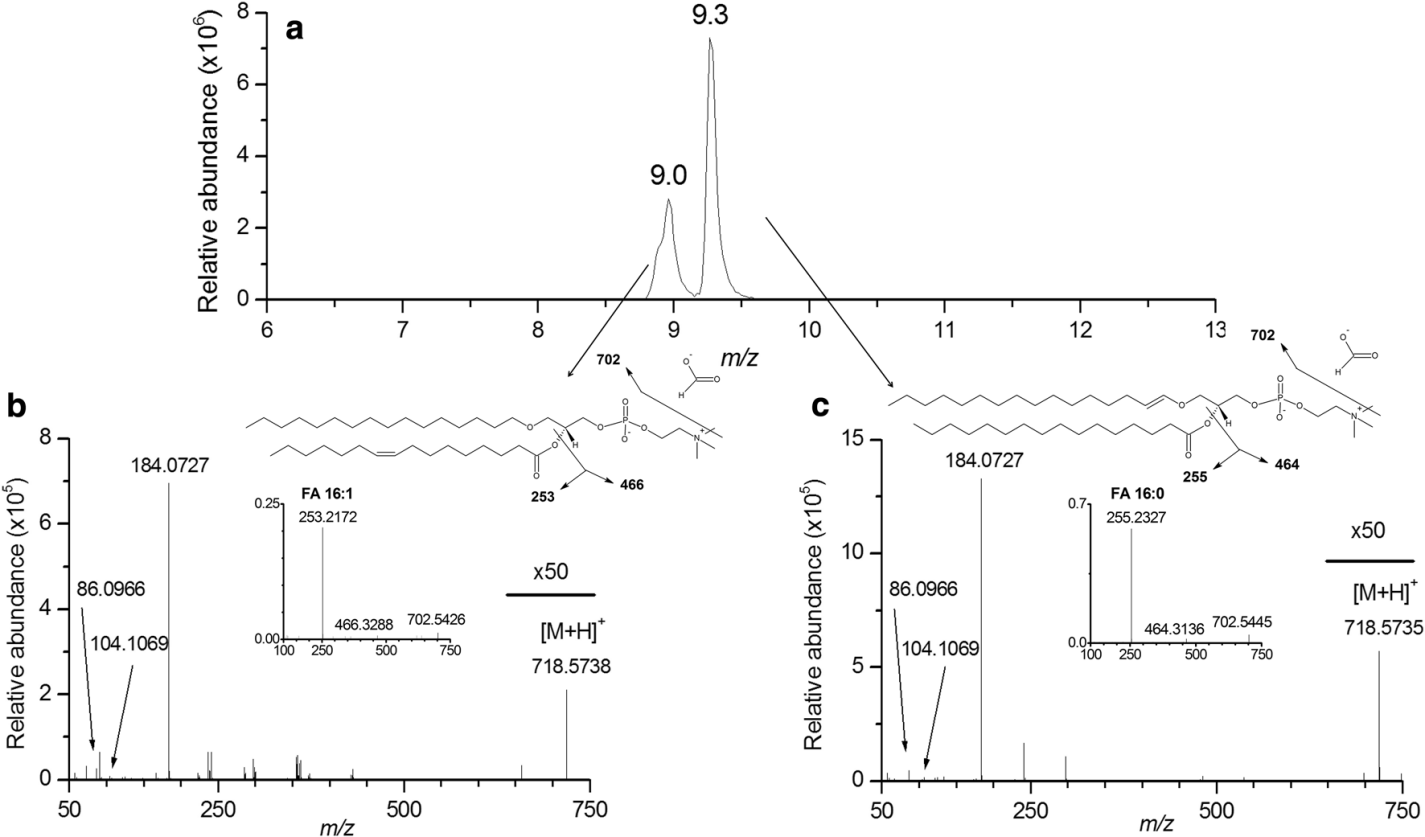
Extracted ion chromatograms of two isomeric lipid species obtained at 9.0 and 9.3 min (**a**). CID mass spectra of these two ions in the positive ionization mode shows the same fragmentation pattern, with the characteristic fragment of phosphocholine at *m/z* 184.0727. Additional CID spectra performed in the negative ionization mode enabled to determine the presence of the fatty acids FA 16:1 (*m/z* 253.2172) (**b**) and FA 16:0 (*m/z* 255.2327) (**c**), corresponding to two distinct PC species, namely alkyl–acyl-PC 32e:1 and alkenyl–acyl-PC32p:0

The characterization of isomeric lipid species constitutes another critical issue, not only of “shotgun lipidomic approaches”, but also of hyphenated liquid chromatography coupled mass spectrometry. This issue can be solved for TG lipid species by increasing collision energy to refine their structural characterization (Bird et al. 2011), and also for a few other lipid species such as particular glycerophospholipid species containing alkyl or alkenyl fatty acyl moieties. For example, two kinds of annotations are proposed for the protonated [M + H]^+^ ions detected at *m/z* 718.5738 and *m/z* 718.5535, at 9.0 and 9.3 min respectively: PC32e:1(alkyl–acyl) or PC32p:0(alkenyl-acyl) (Fig. 5a). Structural characterizations of these lipid species were achieved by performing MS/MS experiments on [M + H] ^+^ and [M − H + CHO_2_]^−^ (*m/z* 740.5550 and 740.5556) ions in positive and negative ion modes, respectively. Only fragmentation experiments in the negative ion mode enabled to distinguish between the alkyl-acyl form PC32e:1 by the presence of the FA 16:1 (*m/z* 253.2172) at 9.0 min (Fig. 5b) and the alkenyl-acyl form PC32p:0 by the presence of the FA 16:0 (*m/z* 255.2327) at 9.3 min (Fig. 5c).

Finally, informative MS/MS experiments led to the confirmation of 418 out of the 771 annotated lipid species, corresponding to 304 unique lipid species. These 771 annotated lipid species are displayed in the supplementary material section (Table S4). Table S4 also displays their exact masse, retention time, mass accuracy, and RIA errors. It also includes intra-assay precision results obtained for each lipid species in QC samples, fragment ions observed during MS/MS experiments, and at last two proposed level of annotation: (i) exact mass, retention time and validated MS/MS experiments (level a) and (ii) exact mass and retention time (level b). This list constitutes to our knowledge the largest database of unique lipid species found in human CSF.

Unlike metabolomics, very few standards of lipid species are available compare to the huge amount of endogenous lipid species present in complex biological matrices. According to the metabolomics standards initiative (MSI), annotations in untargeted lipidomic studies correspond to putatively annotated compounds (level 2 of identification) or putatively characterized compound classes (level 3 of identification) (Sumner et al. 2007). In addition, the high level of structural precision provided by public lipid databases cannot be achieved using a single analytical method, (e.g., double bonds localization or cis–trans isomerism) and without appropriates internal standards.

When compared to previous studies conducted in human CSF samples using ESI mass spectrometry, most of the major lipid families such as glycerophospholipids (Mudler et al. 2003; Kosicek et al. 2010; Kosicek et al. 2012; Fonteh et al. 2013), sphingomyelins, sterols and free fatty acids (Tourtellotte 1959), that are usually analyzed by several complementary targeted methods, were detected in a single experiment thanks to our global approach. Sphingolipids, which represent less than 20 % of total lipid species in CSF, are of particular importance in the context of biomarker discovery for neurological diseases, such as lysosomal or unknown neurometabolic diseases. Numerous rare diseases involving the different steps of sphingolipid biosynthesis pathway, from de-novo synthesis of ceramides to the biosynthesis of complex lipids such as gangliosides and sulfatides, have already been described (Han et al. 2003; Kolter and Sandhoff 2006; Gu et al. 2008; Mielke et al. 2010; Fan et al. 2013). As no lists of lipid species were provided in these studies, it is unfortunately not possible to compare the number and nature of lipid species detected using our method to those obtained with the previous ones. Furthermore, in addition to the annotated lipid species, more than 20,000 features remain unannotated but are analytically relevant for statistical analysis in the frame of an untargeted lipidomic strategy.

## Conclusion

We have developed and validated an untargeted lipidomic approach enabling to profile, annotate and characterize numerous lipid species, including isobars and isomers, in human CSF samples. Our method enabled the detection of more than 30,000 reliable features suitable for statistical analysis, among which 550 were annotated as unique monoisotopic lipid species using annotation tools. Thanks to tandem mass spectrometry experiments, 304 annotations of distinct lipid species were improved in both positive and negative ion modes. Finally, our study provides an extensive database of the human CSF lipidome which is available to the scientific community. It constitutes a relevant foundation for future studies aimed at finding biomarkers of neurological disorders.

## Electronic supplementary material

Below is the link to the electronic supplementary material.

Supplementary material 1 (DOCX 72 kb)

Supplementary material 2 (XLSX 158 kb)

Supplementary material 3 (XLSX 1418 kb)
