# Supplementary material for: Annotation of the human cerebrospinal fluid lipidome using high resolution mass spectrometry and a dedicated data processing workflow
Source: Metabolomics. 2016 Apr 7;12:91. doi: 10.1007/s11306-016-1023-8 (PMC4824825; doi:10.1007/s11306-016-1023-8)
Supplement: Supplementary file 1 — Supplementary material 1 (DOCX 72 kb) [file 11306_2016_1023_MOESM1_ESM.docx]

**Supplementary Material**

**Annotation of the human cerebrospinal fluid lipidome using high resolution mass spectrometry and a dedicated data processing workflow.**

Alexandre Seyer^1^, Samia Boudah^2^, Simon Broudin^1^, Christophe Junot^2^ and Benoit Colsch^2#^

^1^ Profilomic SA, Boulogne-Billancourt, France.

^2^ CEA-Saclay, DSV/iBiTec-S/SPI, Laboratoire d’étude du Métabolisme des Médicaments, MetaboHUB-Paris, Gif-sur-Yvette, France.

**AUTHOR INFORMATION**

# Benoit Colsch

Phone: +33 1 69 08 73 50

Fax: +33 1 69 08 59 07.

E-mail: [benoit.colsch@cea.fr](mailto:benoit.colsch@cea.fr)

**TABLES**

[**Table S1.** Lipid composition of the internal standard mix (at 10x concentration). **4**](#_Toc441494793)

[**Table S2.** List of parameters used for the XCMS package software. **5**](#_Toc441494794)

[**Table S3.** Extraction recoveries and repeatability (n=5) for each internal standard category. **6**](#_Toc441494795)

**Table S4.** List of lipid species annotations obtained from human CSF samples **(in a separate excel file**). This list includes all parameters such as mass-to-charge ratio, retention time (min), mass deviation (ppm), relative isotopic abundance error (%), specific retention time windows for each lipid class, chromatographic peak area, coefficient of variation in QC samples and coefficient of correlation in diluted QCs along with parent and fragment ions observed in MS/MS experiments. In addition, two level of annotation are proposed, level a: exact mass, retention time and validated MS/MS experiments and level b: exact mass and retention time.

**Table S5.** In-silico lipid database used in this study along with reference ions for each lipid class/subclass (**in a separate excel file**).

**FIGURES**

[**Figure S1.** Comparison chromatographic peak areas of 162 lipid species from a CSF total lipid extract, selected by QuantBrowser *via* a processing method (ICIS algorithm) and detected automatically by XCMS (CentWave). **7**](#_Toc441494796)

[**Figure S2.** Distribution of the absolute deviation (%) of the relative isotopic abundance (RIA) of endogenous lipid species detected and annotated (black square) and of internal standards (red square). **8**](#_Toc441494797)

# **Table S1.** Lipid composition of the internal standard mix (at 10x concentration).

# Table S2: List of parameters used for the XCMS package software.

| findPeaks |  |
| --- | --- |
| method | centWave |
| peakwidth | 15-40 |
| mzTol | adjusted to sample |
| snthresh | adjusted to sample |
| noise | 500 |
| prefilter | 5-1000 |
| mzdiff | -0.001 |
| groupA |  |
| minfrac_GA | 0 |
| minsamp_GA | 0 |
| bw_GA | 5 |
| mzwid_GA | 0.005 |
| max_GA | 50 |
| retcorr |  |
| method | loess |
| missing | 1 |
| extra | 1 |
| span | 0.2 |
| family | gaussian |
| groupB |  |
| minfrac_GB | 0 |
| minsamp_GB | 0 |
| bw_GB | 5 |
| mzwid_GB | 0.005 |
| max_GB | 50 |

# Table S3. Extraction recoveries and repeatability (n=5) for each internal standard category.

# Figure S1. Comparison chromatographic peak areas of 162 lipid species from a CSF total lipid extract, selected by QuantBrowser *via* a processing method (ICIS algorithm) and detected automatically by XCMS (CentWave).

# Figure S2. Distribution of the absolute deviation (%) of the relative isotopic abundance (RIA) of endogenous lipid species detected and annotated (black square) and of internal standards (red square).
